# Molecular insights into the protective role of nano-naringin in mancozeb-induced testicular damage and infertility

**DOI:** 10.1186/s40659-026-00712-y

**Published:** 2026-07-06

**Authors:** Eman I. Hassanen, Sara S. Elbagwry, Zienab E. Eldin, Rehab A. Azouz, Marwa A. Ibrahim, Rawhia Doghaim

**Affiliations:** 1 Pathology Department, Faculty of Veterinary Medicine, Cairo University, Giza, 12211 Egypt; 2 Pathology Department, Faculty of Veterinary Medicine, Egyptian Chinese University, Cairo, 19346 Egypt; 3 Materials Science and Nanotechnology Department, Faculty of Postgraduate Studies for Advanced Science, Beni-Suef, 62511 Egypt; 4 Toxicology and Forensic Medicine Department, Faculty of Veterinary Medicine, Cairo University, Giza, 12211 Egypt; 5 Biochemistry and Molecular Biology Department, Faculty of Veterinary Medicine, Cairo University, Giza, 12211 Egypt

**Keywords:** Chitosan nanoparticles, Gene expression, Mancozeb, Naringin, Oxidative stress, Reproductive toxicity

## Abstract

Mancozeb (MZ) is an extensively used fungicide with well-documented reproductive toxicity induced by oxidative stress and hormonal disruption. Antioxidants such as naringin (NAR) have been studied as protective agents, but their therapeutic application is limited by poor bioavailability. Nano-formulated approaches, including chitosan-coated nanoparticles (NARNPs), offer a promising strategy to enhance delivery and efficacy. The present study explores this concept by evaluating the protective effects of NAR and NARNPs against MZ-induced testicular damage and infertility in rats. 42 adult male albino Wistar rats were divided into six groups (n = 7) as follows: (1) control, (2) NAR (20 mg/kg bwt), (3) NARNPs (20 mg/kg bwt), (4) MZ (250 mg/kg bwt), (5) NAR + MZ, and (6) NARNPs + MZ. Data revealed that the exposure of rats to MZ caused a significant decrease in the body weight gain, sperm viability and motility, serum testosterone level, and testicular antioxidant enzyme activities, along with an increase in the lipid peroxidation. MZ also inhibited the expression of steroidogenic genes and proliferating markers in testes. The administration of NAR with MZ partially restored oxidative homeostasis and the reproductive biomarkers. Otherwise, NARNPs were more effective than NAR, which substantially improved the testicular morphology and function, as well as partially returned the gene expression back to normal with higher immunoexpression of proliferating markers in testicular tissues. The study highlights the potential of nano-formulated NAR as a promising strategy to counteract MZ-induced reproductive damage and improve testicular health via enhancing steroidogenic gene expression.

## Introduction

Fertility is a fundamental aspect of animal health and productivity. It plays a critical role in maintaining the health, productivity, and proper management of animals, whether pets or farm animals [58]. In veterinary medicine, reproductive efficiency has a direct impact on the economic returns, especially in livestock production and breeding industries [79]. Fertility challenges can lead to reduced productivity, increased veterinary costs, and significant economic losses for farmers, breeders, and pet owners [26].

Mancozeb (MZ), an organometallic polymeric complex of manganese/zinc ethylene-bisdithiocarbamate fungicide, is widely employed in agriculture to protect crops from fungal diseases [4]. MZ is classified as a harmful fungicide due to its considerable toxic impact on mammals, fish, birds, and the environment [27, 40]. Agricultural workers and those handling chemicals face the highest risk of exposure, which can occur through skin contact, inhalation of fine particles or spray, or accidental ingestion, such as eating or smoking without prior handwashing [1]. However, concerns have been raised regarding MZ’s potential impact on reproductive health [68]. MZ has an important health impact due to its ability to trigger oxidative stress in biological systems, while exposure to this compound disrupts cellular redox homeostasis by increasing the production of reactive oxygen species (ROS) and diminishing essential antioxidant enzymes [81, 82]. Moreover, this oxidative imbalance contributes to cellular damage through mechanisms such as lipid peroxidation, protein modification, and DNA degradation, with particularly harmful effects on important organs like the testes [56]. Many earlier experimental studies revealed that MZ could alter both spermatogenesis and steroidogenesis alongside oxidative stress-mediated testicular damage in adult rats [50, 75].

Naringin (NAR), 7-hydroxyflavanone 7-O-beta-D-glucoside, is a natural compound found in citrus fruits, particularly grapefruit, where it contributes to the characteristic bitter taste of fruits [23]. Several studies have highlighted that NAR has multiple biological properties, including antioxidant, anti-inflammatory, anti-ulcer, anti-osteoporotic, anti-apoptotic, and anti-carcinogenic activities [39, 46, 60]. However, NAR has been documented to exhibit low oral bioavailability [76]. This limitation is primarily attributed to its poor solubility and limited absorption in the gastrointestinal tract, resulting in poor absorption into the bloodstream and reducing its therapeutic effectiveness [44]. Various methods can be employed to overcome this limitation, including co-crystallization, solid dispersions, amorphous carriers, and coating by natural polymers. [25, 44].

Chitosan, a natural biopolymer derived from crustacean exoskeletons, has a diversity of biological properties due to its non-toxic nature, high biocompatibility, biodegradability, and its ability to form stable nanoparticles that make it used as a drug delivery system [18]. Chitosan-based drug delivery system provides a promising and adaptable approach to modern therapeutics because of their important physical, chemical, and biological properties [35]. One of its favorable benefits is drug release control, while it can be released slowly at the target site [67]. Therefore, both the drug dose and frequency of administration could be reduced, achieving more pharmaceutical activity with fewer side effects. It also provides controlled and sustainable release of oral medication due to its mucoadhesive properties, hence improving drug accumulation where it’s needed most [29]. Moreover, low toxicity and high biocompatibility are also the two major advantages of Cs coating, which allow repeated use without causing tissue irritation or immunological reactions [78]. Furthermore, Cs coatings aid in bypassing gastrointestinal tract barriers like intestinal proteases, gastric acid, and saliva enzymes, which normally break down or inactivate many medicinal pharmaceuticals such as NAR [42]. All these features collectively make Cs NPs a valuable platform for improving drug stability, efficacy, and patient compliance across a range of biomedical applications [72].

The main goal of the current study is to establish the novel contribution of nano-formulated NAR in reproductive toxicity research by directly comparing its protective efficacy against MZ-induced testicular damage with conventional flavonoid approaches previously reported. The use of Cs NPs encapsulation to enhance biological activity and targeting is another novel aspect, especially in the context of protecting reproductive organs. Additionally, the potential mechanistic way of NAR was investigated, interpreted as associations rather than definitive causal mechanisms, by several measurements such as major male sex hormone levels, semen analysis, oxidant/antioxidant balance, the transcript levels of major steroidogenesis-regulating genes, histopathological examination of the testis, and immunoexpression of major proliferating markers, including DOG-1 and PCNA. Furthermore, the successful encapsulation of NAR core with Cs coating was evaluated by the encapsulation efficiency and loading capacity, along with other characterization methods.

## Materials and methods

### Chemicals

The study was conducted using a commercial fungicide formulation of MZ (98%WP, manganese ethylenebis, Dithiocarbamate, polymeric, complex with zinc salt) with chemical formula (C8H12MnN4S8Zn). This formulation was obtained from Dow AgroSciences, France. It was dissolved in sesame oil to achieve the desired concentration. Naringin (purity ≥ 98%), named by CAS number 10236–47-2, was obtained from Santa Cruz Biotechnology, USA. It was dissolved in methanol and later diluted with deionized distilled water to obtain the required dosage. Chitosan (Cs) (degree of deacetylation > 90%, molecular weight 200–300 kDa) was obtained from Sigma-Aldrich (St. Louis, MO, USA). Glacial acetic acid (99.7%), sodium tripolyphosphate (TPP, > 98%), absolute ethanol (99.5%), and other analytical-grade chemicals were supplied by Merck KGaA (Darmstadt, Germany). Deionized water was used throughout all experiments. Phosphate-buffered saline (PBS, pH 7.4) was prepared in-house using Merck reagents.

### Preparation and characterization of nanoparticles

#### Preparation of chitosan nanoparticles

Blank chitosan nanoparticles were synthesized using the ionotropic gelation method, which relies on the electrostatic interaction between the cationic amino groups of chitosan and the polyanionic crosslinker TPP [11]. Briefly, 1 mg/mL of Cs was dissolved in 100 mL of 1% (v/v) aqueous acetic acid by stirring at 600 rpm overnight at room temperature, yielding a clear, homogeneous viscous solution. The solution was filtered through a 0.45 μm syringe filter (Sartorius, Germany) to remove undissolved particulates and stored at 4 °C. A 1% (w/v) TPP solution was prepared in deionized water. For nanoparticle formation, 20 mL of the TPP solution was added dropwise to 50 mL of the chitosan solution under vigorous stirring at 800 rpm over 30 min. The suspension was further stirred at 800 rpm for an additional 60 min to ensure complete nanoparticle formation and cross-linking. Nanoparticles were collected by centrifugation at 15,000 rpm for 30 min at 4 °C using a Sigma 3–30KS centrifuge (Sigma Laborzentrifugen, Germany). The pellet was washed three times with deionized water to remove residual TPP and unreacted materials. The resulting nanoparticles were lyophilized using a Labconco FreeZone 6 Freeze Dry System (Labconco, USA) at − 50 °C for 48 h to yield a dry, powdery product suitable for further analysis.

#### Preparation of naringin-loaded chitosan nanoparticles

NARNPs were prepared using the ionotropic gelation technique [36]. Briefly, 1 mg/mL NAR was dissolved in 10 mL of absolute ethanol via ultrasonication for 15 min. Separately, 1 mg/mL Cs was dissolved in 100 mL of 1% (v/v) aqueous acetic acid by stirring at 600 rpm overnight at room temperature. The Cs solution was filtered through a 0.45 μm syringe filter and stored at 4 °C. For nanoparticles formation, 5 mL of the NAR solution was added dropwise to 50 mL of the Cs solution under vigorous stirring at 800 rpm for 24 h. Subsequently, 20 mL of a 1% (w/v) TPP solution was added dropwise over 30 min while keeping stirring. The mixture was further stirred at 800 rpm for an added 1 h to ensure complete cross-linking and nanoparticle formation. Nanoparticles were collected by centrifugation at 15,000 rpm for 30 min at 4 °C, washed three times each with deionized water and absolute ethanol, and lyophilized using a Labconco FreeZone 6 Freeze Dry System at − 50 °C for 48 h.

#### Characterization of the prepared nanoparticles

The crystalline structure and phase change of Cs NPs and NARNPs were analyzed using a powder X-ray diffractometer (Bruker D8 Advance, Bruker Corporation, Germany) with Cu Kα radiation (λ = 1.5406 Å) at 40 kV and 40 mA. Diffraction patterns were recorded over a 2θ range of 5–60° at a scanning rate of 0.02° per second. Particle size distribution, polydispersity index (PDI), and zeta potential were measured by the dynamic light scattering (DLS) and electrophoretic light scattering using a Malvern Zetasizer Nano ZS (Malvern Instruments, UK). Lyophilized nanoparticles were dispersed in deionized water (1 mg/mL) and sonicated for 5 min prior to measurement. Analyses were performed at 25 °C and a scattering angle of 173°, with all measurements conducted in triplicate. The morphology of NARNPs was analyzed using transmission electron microscopy (TEM; JEM-1400, JEOL, Japan). To decide loading capacity (LC) and entrapment efficiency (EE), 10 mg of lyophilized NPs were dispersed in 10 mL of 1% (v/v) acetic acid, sonicated for 30 min, and centrifuged at 10,000 rpm for 15 min. The supernatant was analyzed spectrophotometrically at 282 nm [57]. Then, LC and EE were calculated as follows:

$$ \begin{gathered} LC\left( \% \right) = \left( {{\text{mass of NAR encapsulated in NPs/mass of NPs}}} \right){\text{ }} \times {\text{ }}100 \hfill \\ EE\left( \% \right) = \left( \begin{gathered} {\text{mass of NAR encapsulated in}} \hfill \\ {\text{nanoparticles/initial mass of NAR used}} \hfill \\ \end{gathered} \right) \times 100 \hfill \\ \end{gathered} $$

### Animals and experimental design

All experimental procedures and protocols were conducted following the ARRIVE guidelines and approved by the Institutional Animal Care and Use Committee (IACUC) of Cairo University (Approval No. Vet CU131020241057).

Forty-two adult male albino Wistar rats (weighing 150 ± 20 g, aging 8–10 weeks old) were obtained from the Lab. Animal Housing of VACSERA, Helwan, Egypt. The sample size was statistically determined using the power calculation method. The animals were kept in plastic cages and provided with standard commercial pelleted feed along with ad libitum access to water throughout the experimental period. Before the experiment, the rats underwent a two-week acclimatization period to adjust to the laboratory environment, during which their health status was checked. After the acclimation period, the body weight of rats increased to 170–200 g (10–12 weeks old), marking the beginning of the experiment at a weight suitable for fertility assessment based on previously published research [41, 55].

The rats were randomly assigned into six groups (n = 7) and received the following substances daily by oral gavage for 54 days. Group (1) served as the negative control and was given sesame oil. Group (2) was given NAR at a dose of 20 mg/kg bwt. While group (3) was given NARNPs at the same dose. Group (4) received MZ at 250 mg/kg bwt, corresponding to 1/20 of its oral LD50. Groups (5) and (6) co-administered MZ with either NAR or NARNPs, respectively, at the same previously mentioned doses. Mancozeb was administered after NAR and NARNPs, with a time interval of 2 h. The pesticide dose was selected based on its documented LD50 of 5000 mg/kg bwt [80], while the NAR dose was chosen from prior studies [48]. All animals were monitored daily for any clinical signs and mortalities.

### Sampling

After 54 days postdosing, the rats were anesthetized using an intramuscular injection of xylazine (10 mg/kg) and ketamine (90 mg/kg), and then blood samples were collected from the orbital sinus. Blood samples were centrifuged at 3500 rpm for 5 min to separate clear serum, which was then stored at − 21 °C for further biochemical analysis. Following this, the rats were euthanized by decapitation, and testis were obtained. The left testis was stored at − 80 °C till used for oxidative stress assessment and molecular analysis, while the right testis was fixed in 10% neutral buffered formalin till used for histopathological and immunohistochemical examination.

### Semen analysis

The cauda epididymis of the testis was removed and placed in 2 mL of sodium chloride 0.9% solution in a sterilized petri dish at 37 °C. Then, a sterilized scissor was used to get the epididymal contents out into the solution to form a suspension. The sperm count was performed under a light microscope at 100x, while the sperm motility and morphology were examined at a magnification of 400x. About 100 spermatozoa were randomly seen under an oil immersion lens in several fields to measure the percentage of sperm abnormalities [28].

### Hormonal assay

Serum concentrations of testosterone were measured according to the instructions provided with the manufacturer’s kits (Biodiagnostic, Cairo, Egypt).

### Oxidative stress evaluation

The collected frozen tissue samples from the testis (0.1 g) were homogenized in 1 mL cold buffer (50 mM potassium phosphate buffer with 1 mM EDTA, pH 7) using a glass porcelain homogenizer for 5 min. Then the homogenates were centrifuged at 7000 xg for 15 min according to the method described by [34]. All processes occurred at 4 ^O^C to estimate some oxidants and antioxidant markers. Lipid peroxidation, Malondialdehyde (MDA), levels in the testis tissues were measured according to the method of Satoh, [70]. Additionally, the Glutathione reductase (GR) and catalase activities (CAT), were estimated in testis homogenate according to the methods described by Beutler, [17] and Aebi, [6], respectively. All measurements were performed in accordance with the manufacturer’s instructions for the acquired kits (Biodiagnostic Com. Egypt).

### Quantitative RT-PCR analysis for steroidogenic regulated genes

Following the manufacturer’s guidelines, the total RNA was isolated using the easy-BLUETM Total RNA Extraction Kit from iNtRON Biotechnology (CAT. No.17061). For cDNA synthesis, the SenScript TM RH (-) cDNA Synthesis Kit was employed, also from iNtRON Biotechnology (CAT. No. 25011). Gene expression levels were quantified compared to a control using the StepOnePlus TM Real-Time PCR System with SYBR-Green master mix (Thermo Scientific CAT. No. 4309155). The primers specific to each gene are listed in Table 1. The PCR cycling protocol involved 40 cycles: 10 s at 95 °C for denaturation, 15 s at 58 °C for annealing, and 15 s at 72 °C for extension. A melting curve analysis was performed afterward to verify the specificity of the amplification, starting with 15 s at 95 °C, then 1 min at 60 °C, with data collection at 0.3 °C intervals up to 95 °C. The relative quantification of gene expression was calculated using the modified fold change method, normalizing the data against the β-actin housekeeping gene. Each experiment included no-template controls for each gene, and duplicate reactions were performed for each sample.

**Table 1:** Primer sets for the studied genes

	Sense	Antisense	Ampli-con	Accession no	References
*Star*	TGGCTGCCAAAGACCATCAT	TGGTGGGCAGTCCTTAACAC	241	NM_031558.3	[5]
*Cyp19a1*	TGACGTCACTGACAACTCGG	CAAGTCCACGACAGGCTGAT	235	NM_017085.2	[15]
*Hsd-3β*	CTCACATGTCCTACCCAGGC	TATTTTTGAGGGCCGCAAGT	362	NM_001007719.3	[53]
*Actb*	CCGCGAGTACAACCTTCTTG	CAGTTGGTGACAATGCCGTG	297	NM_031144.3	[3]

Cytochrome P450-19A1 (Cyp19a1), 3β-hydroxysteroid dehydrogenase (HsdD-3β), Steroidogenic Acute Regulatory protein (Star), and beta-actin housekeeping gene (Actb)

### Histopathological examination

Tissue samples preserved in formalin were processed through a graded series of alcohol and xylene for dehydration and clearing. The tissues were then embedded in paraffin, sectioned at 4.5μm thickness using a microtome, and stained with hematoxylin and eosin (H&E) for histological examination. Microscopic analysis was conducted using an Olympus BX43 light microscope, and digital images were captured with an Olympus DP27 camera, which was integrated with Cell-Sens Dimensions software (Version 1.13; Core Version XV 3.12, Build 13,479) [16].

The Johnsen scoring system was used to evaluate spermatogenesis across experimental groups. About 50 seminiferous tubules per group were graded on a scale from 1 to 10, based on the most advanced germ cell type present and the degree of spermatogenic organization, with higher scores indicating more complete and organized spermatogenesis, while the lowest score indicates empty tubules [45]. Additionally, some histomorphometric assessments were conducted using TSView software, including measurement of seminiferous tubular diameter and the thickness of the germ cell layers.

#### Immunohistochemical localization of some proliferating markers within testes

Paraffin-embedded testicular tissues were sectioned at 4–5µm and mounted on positively charged slides. Sections were deparaffinized with xylene, rehydrated through descending ethanol concentrations, and subjected to heat-induced epitope retrieval using citrate buffer (pH 6.0). Endogenous peroxidase activity was blocked with 3% hydrogen peroxide, followed by incubation with 5% bovine serum albumin to prevent nonspecific binding. Primary antibodies, including mouse monoclonal anti-PCNA and rabbit monoclonal anti-DOG-1, were applied according to manufacturer recommendations. After washing, an HRP-conjugated secondary antibody was added, and staining was visualized using 3,3-diaminobenzidine (DAB), producing a brown precipitate at antigen sites. Hematoxylin counterstaining was performed for structural context. Finally, sections were dehydrated, mounted, and examined under a light microscope (Olympus BX43) with images captured by an Olympus DP27 camera linked to CellSens Dimensions software, to assess PCNA nuclear staining and DOG-1 cytoplasmic/membranous localization.

A semiquantitative total scoring system was conducted by calculating the mean percentage of positive cells and the intensity of DAB staining across groups. The mean percentage of positive cells was calculated by comparing a visual approximation of the cells that stained positive for DOG1 or PCNA with the total number of germ cells present on well-known measured area. A grading scale from 0 to 4 was used as follows: (0) negative reaction, (1) less than 25% positive cells, (2) from 25 to 50% positive cells, (3) from 50 to 75% positive cells, (4) more than 75% positive cells. While the DAB-staining intensity was evaluated using a four-grading scale (0–3) as follows: (0) negative, (1) weak, (2) moderate, (3) strong. Finally, the total immunoreactivity score for each immune marker was calculated by adding the proportion score (mean percentage of positive cells) and the intensity score [32, 38].

#### Statistical analysis

Statistical analysis was performed using SPSS software (version 27.0; SPSS Inc., Chicago, IL, USA). Power analysis was conducted using G-Power software (version 3.1.9.7) to determine the appropriate sample size for a one-way fixed-effects independent ANOVA across six groups. Sperm concentration was designated as the primary outcome measure. A large expected effect size (f = 0.60) is used due to the expected significance difference between Groups (5) and (6), a significance level P of 0.05, and a target statistical power of 0.80. The minimum required sample size was calculated to be forty-two rats with seven rats per group. Differences between groups were evaluated using one-way analysis of variance (ANOVA), followed by Tukey’s post hoc test for multiple comparisons. Moreover, data for pathological scoring were analyzed using Kruskal–Wallis H test and Mann–Whitney U test. *P* ≤ *0.05* is considered statistically significant. The results were presented as means ± standard error of means (SEM).

## Results

### Characterization of the prepared nanoparticles

**Fig. 1:**
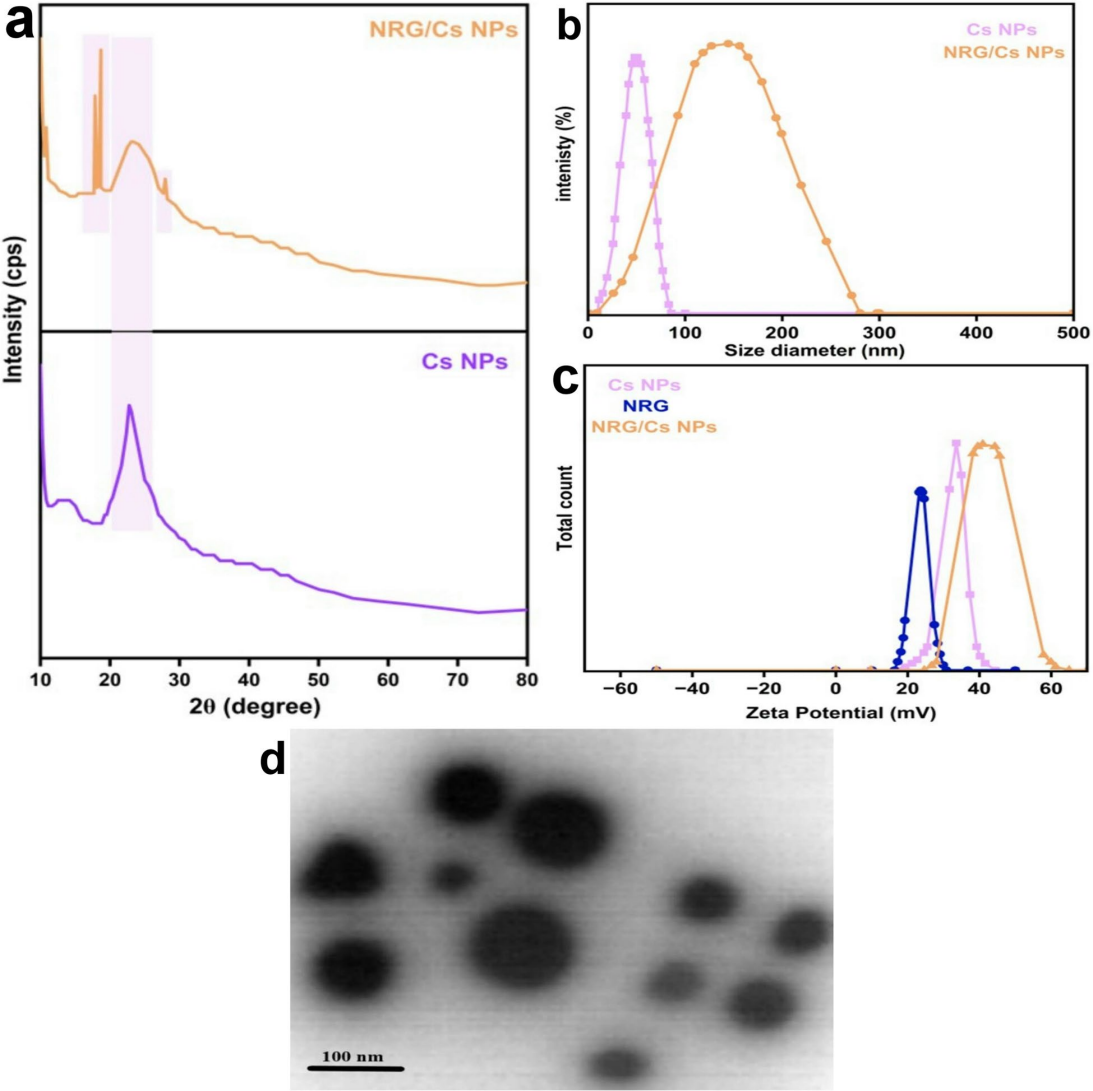
Characterization of the prepared nanoparticles compared with the individual material. **a** XRD pattern of Cs NPs and NRG/Cs NPs (NAR NPs). **b** Particle size analysis of Cs NPs and NRG/Cs NPs (NAR NPs). **c** Zeta potential of Cs NPs, NRG, and NRG/Cs NPs (NAR NPs). **d** TEM image of NAR NPs at a scale bar of 100 nm

As shown in Fig. 1a, the XRD pattern of pristine Cs NPs showed a broad diffraction peak 2θ = 20–23° with the absence of sharp distinct peaks suggests a low degree of crystallinity in the synthesized Cs NPs. Significant changes were seen in the XRD pattern of NARNPs. The broad peak characteristic of chitosan at 2θ = 20–23^0^ degrees was still present, but its intensity appeared to be reduced and broadened. More importantly, new, sharper diffraction peaks appeared in the NAR nanocomposite pattern, notably around 2θ = 18–19° and 22–23°, along with other less intense peaks. Figure 1b showed a relatively narrow size distribution curve of Cs NPs with a peak intensity 55.8 ± 2.3 nm and the PDI was determined to be 0.2 ± 0.01. Upon the formation of the NAR NPs, a noticeable shift in the hydrodynamic diameter was seen, with the peak intensity increasing to approximately 147 ± 1.2 nm and the PDI was found to be 0.27 ± 0.01. The Cs NPs and NAR alone displayed a positive zeta potential of approximately + 35 ± 2 mV and + 21 ± 1 mV, respectively, while the NAR NPs showed a zeta potential of approximately + 40 ± 1.4 mV, which is slightly higher than that of the pristine Cs NPs (Fig. 1c). The TEM image reveals spherical-shaped particles with an average size 70–150 nm, but the majority of the particles fall within the 80–120 nm range. The particles appeared relatively uniform, although some variations in size are observable. There is no considerable evidence of aggregation, suggesting good colloidal stability under the observed conditions (Fig. 1d). The loading capacity (LC) of NAR NPs, which defines the weight percentage of the drug in the nanoparticles, was determined to be 11.58 ± 1.05%. Concurrently, the entrapment efficiency (EE), representing the proportion of the initial drug successfully encapsulated, was found to be 93.87 ± 1.33%. These results show a highly efficient and effective formulation process.

### Semen analysis

Sperm quality parameters didn’t significantly differ between the NAR, NARNPs, and control groups. In contrast, the MZ group exhibited a significant decrease in sperm viability, concentration, and motility alongside a significant increase in sperm abnormalities compared to the control group. On the other hand, the co-administration of NAR with MZ revealed a significant elevation in sperm viability, concentration, and motility compared with the MZ group, although it is significantly lower than that of the NAR group. Additionally, the sperm abnormalities of the NAR + MZ group showed a significant reduction compared to the MZ group, but were still high if compared with the NAR group. Similarly, combining NARNPs with MZ significantly elevated the viability, concentration, and motility, as well as decreased the abnormalities relative to the MZ group. In contrast to the viability, there was no significant difference in all sperm quality parameters between the NARNPs + MZ group and NARNPs group (Table 2).

**Table 2:** The effect of MZ and/or NAR/NARNPs on sperm quality parameters

	Control	NAR	NARNPs	MZ	MZ + NAR	MZ + NARNPs
Viability (%)	75.2 ± 1.46	74.8 ± 1.98	75.4 ± 1.4	37.6 ± 0.74*	58.8 ± 2.53*^#^	60.4 ± 2.27*^#^
Concentration (× 10^6^/ml)	26.6 ± 1.06	26.4 ± 0.53	27 ± 0.7	8.8 ± 0.76*	19.1 ± 1.5*^#^	28.4 ± 0.74^#^
Motility (%)	70.4 ± 2.2	67.9 ± 2.8	70.9 ± 2.3	28.4 ± 1.3*	51.9 ± 1.4*^#^	71.4 ± 1.07^#^
Abnormality (%)	11.6 ± 0.74	11.6 ± 0.74	10.2 ± 0.66	41.6 ± 2.13*	15 ± 1.09*^#^	9.8 ± 0.58^#^

Values presented as mean ± SEM (n = 7). ⁎ means a significant difference compared to the corresponding control group, while # means a significant difference compared to MZ group at *P* ≤ *0.05*

### Hormonal assay

The data presented in Table 3 didn’t indicate any significant difference in testosterone level between the control, NAR and NARNPs groups. Otherwise, the MZ group showed a significant decrease in testosterone levels compared to the control group. Whereas the administration of NAR, either free or nanoformulation with MZ, significantly increased the testosterone levels compared with the MZ group, but still lower than those of the corresponding control group.

**Table 3:** The effect of MZ and/or NAR/NARNPs on testosterone levels

	Control	NAR	NARNPs	MZ	MZ + NAR	MZ + NARNPs
Testosterone (nmol/L)	6.74 ± 0.19	6.32 ± 0.46	6.14 ± 0.35	2.85 ± 0.84*	4.1 ± 0.03*^#^	5.28 ± 0.43*^#^

Values presented as mean ± SEM (n = 7). ⁎ means a significant difference compared to the corresponding control group, while # means a significant difference compared to the MZ group at *P* ≤ *0.05*

### Oxidative stress evaluation

No statistically significant differences were observed between the NAR- or NARNPs-treated groups and the control group in the measured oxidative stress parameters (MDA, GR, and CAT). The MZ group showed a significant increase in testicular MDA levels alongside a significant decrease in the GR and CAT activity compared to the control group. On the other hand, the co-administration of NAR (either free or nanoformulation) with MZ revealed a significant reduction in testicular MDA levels compared with the MZ group, but still higher than those of the corresponding control groups. Additionally, the GR and CAT activity of the NAR (free or nano) + MZ groups showed a significant elevation compared to the MZ group, but it was still low when compared with the corresponding control groups (Table 4).

**Table 4:** The effect of MZ and/or NAR/NARNPs on some oxidative stress parameters

	Control	NAR	NARNPs	MZ	MZ + NAR	MZ + NARNPs
MDA (nmol/g)	2.74 ± 0.31	3.11 ± 0.16	3.17 ± 0.49	7.9 ± 0.55*	5.6 ± 0.19*^#^	5.7 ± 0.14*^#^
GR (U/g)	3.5 ± 0.43	3.5 ± 0.63	3.1 ± 0.59	0.48 ± 0.16*	1.5 ± 0.28*^#^	1.4 ± 0.25*^#^
Catalase (U/g)	2.33 ± 1.13	2.3 ± 0.08	2.7 ± 0.09	1.11 ± 0.04*	1.68 ± 0.13*^#^	2.08 ± 0.21*^#^

Values presented as mean ± SEM (n = 7). ⁎ means a significant difference compared to the corresponding control group, while # means a significant difference compared to the MZ group at *P* ≤ *0.05*

### Quantitative RT-PCR analysis for steroidogenic regulated genes

**Fig. 2:**
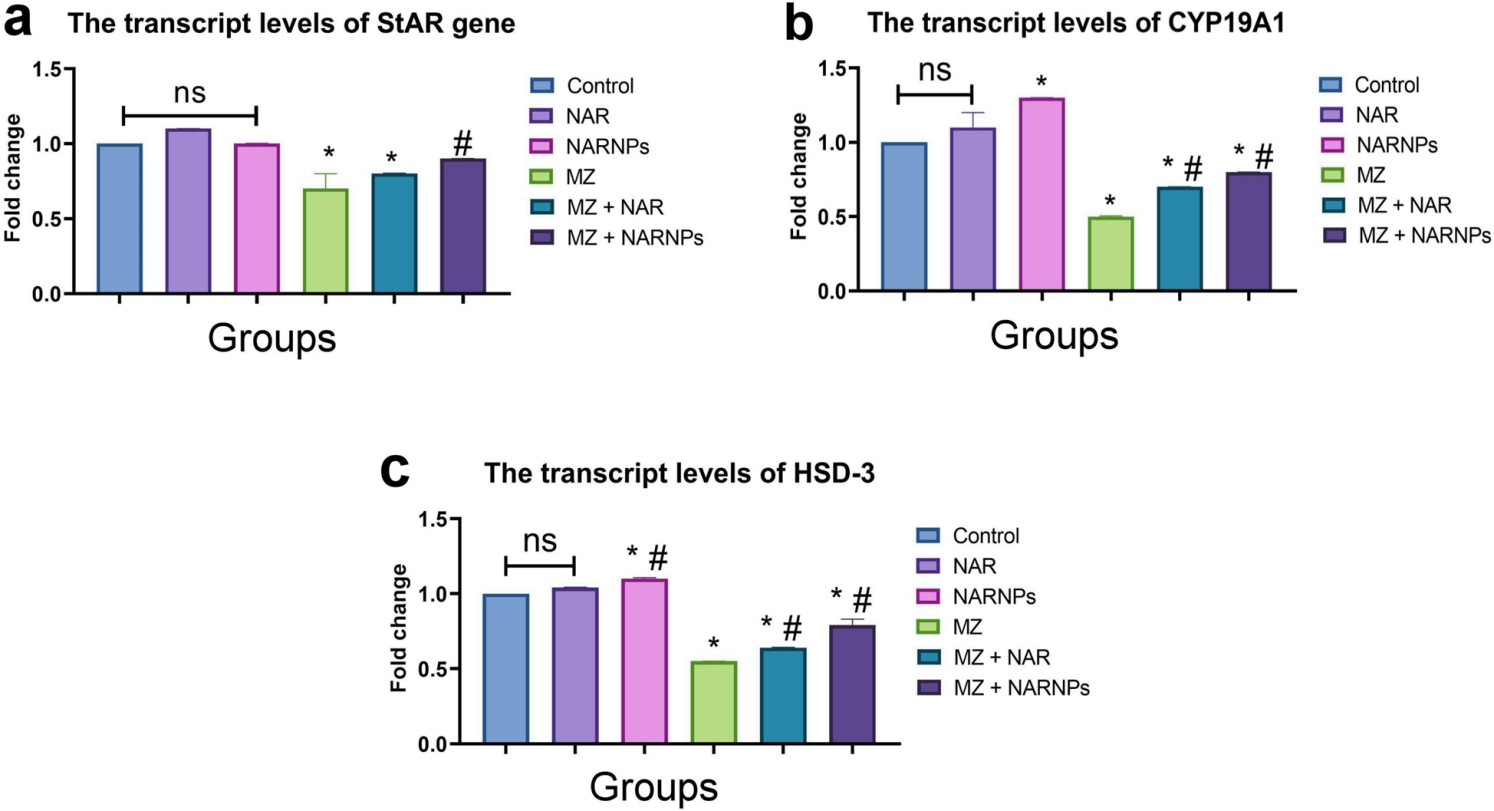
Bar chart represents the testicular mRNA expression levels of StAR **a**, CYP19A1 **b**, and HSD-3β **c** in various experimental groups. Values presented as mean ± SEM (n = 7). ⁎ means a significant difference compared to the corresponding control group, while # means a significant difference compared to MZ group at *P* ≤ *0.05*

The results of gene expressions in either NAR- or NARNPs-treated groups demonstrated no significant changes relative to the control group. MZ administration caused a significant downregulation of the testicular transcript levels of *Cyp19a1, Hsd-3β,* and *StAR* genes. Meanwhile, the co-administration of NAR and NAR NPs with MZ significantly counteracted the negative effects of MZ by upregulating the testicular mRNA expression levels of *Cyp19a1, Hsd-3β,* and *StAR* genes. However, the best improvement seen was in the NAR NPs group compared with the NAR group (Fig. 2).

### Histopathological examination

**Fig. 3:**
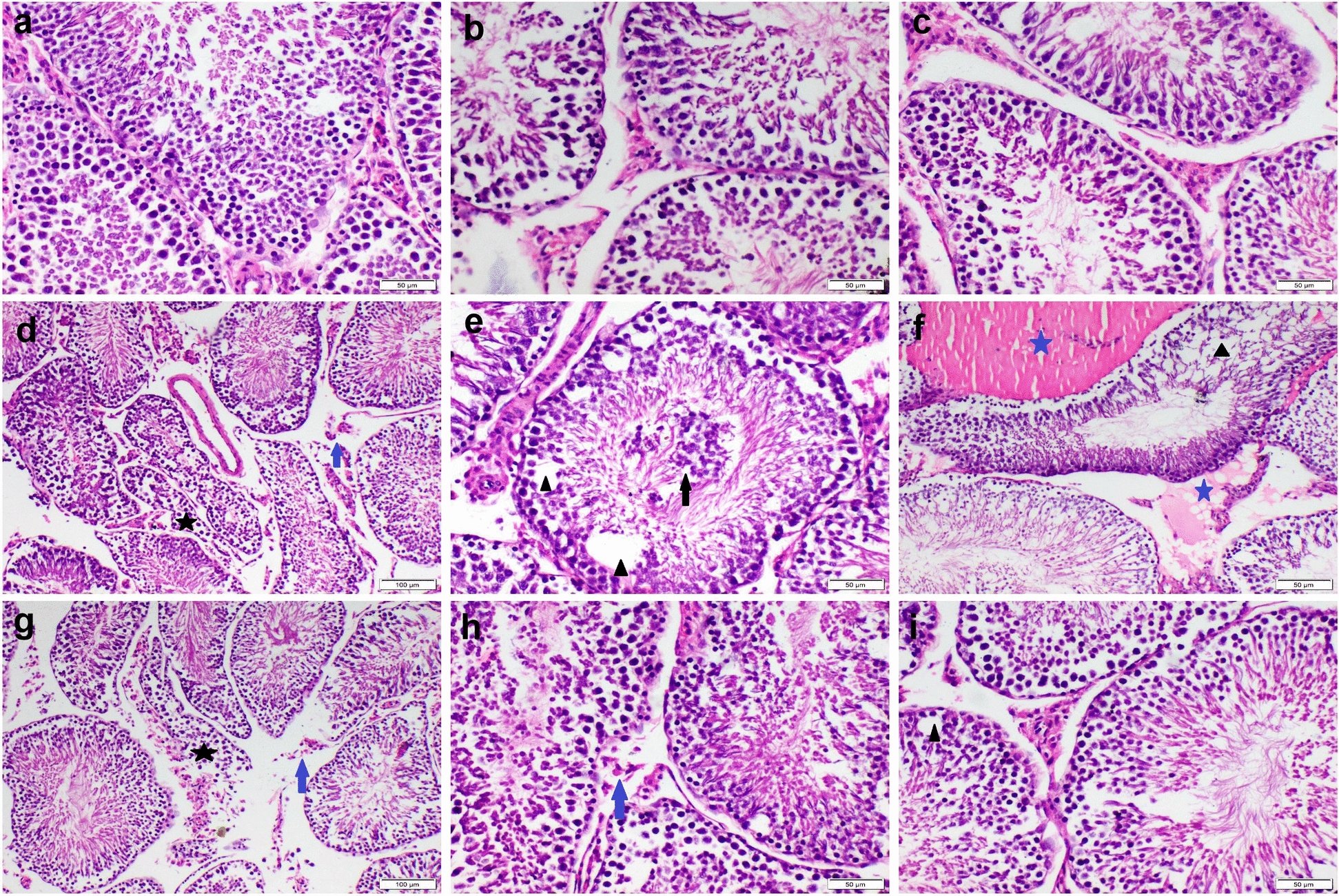
Photographs of H & E-stained testicular sections of various experimental groups. **a** control group, **b** NAR group, **c** NAR NPs group, **d**–**g** MZ group, **h** MZ + NAR group, and **i** MZ + NAR NPs group. Notes: degenerated/atrophied seminiferous tubules (black star), vacuolation (triangle), desquamated germ cells (black arrow), degenerated Leydig cells (blue arrow), edema (blue star). Scale bar 100 µm corresponding to 10 × magnification while scale bar 50 µm corresponding to 20 × magnification

Histological examination of the testes obtained from the control, NAR and NARNPs groups revealed normal architecture of seminiferous tubules with well-organized germinal epithelium. All stages of spermatogenesis were clear, and interstitial Leydig cells appeared morphologically intact. On the other hand, the testes of the MZ group showed marked histopathological alterations, including edema, degeneration, desquamation, and depletion of certain germ cells, mainly spermatogonia and spermatocytes. Some spermatogenic cells showed necrosis manifested by swelling of cells with either pyknotic or lytic nuclei. Interstitial edema, congestion, degeneration, and necrosis of Leydig cells were also clear in some sections. Otherwise, the group administered NAR either free or nanoform with MZ showed restoration of the testicular architecture, but a more pronounced recovery was shown in NARNPs group. Some seminiferous tubules showed mild edema and germinal cell disorganization, while others appeared with normal histological organization. The interstitial tissue showed fewer signs of edema and Leydig cell damage (Fig. 3).

**Fig. 4:**
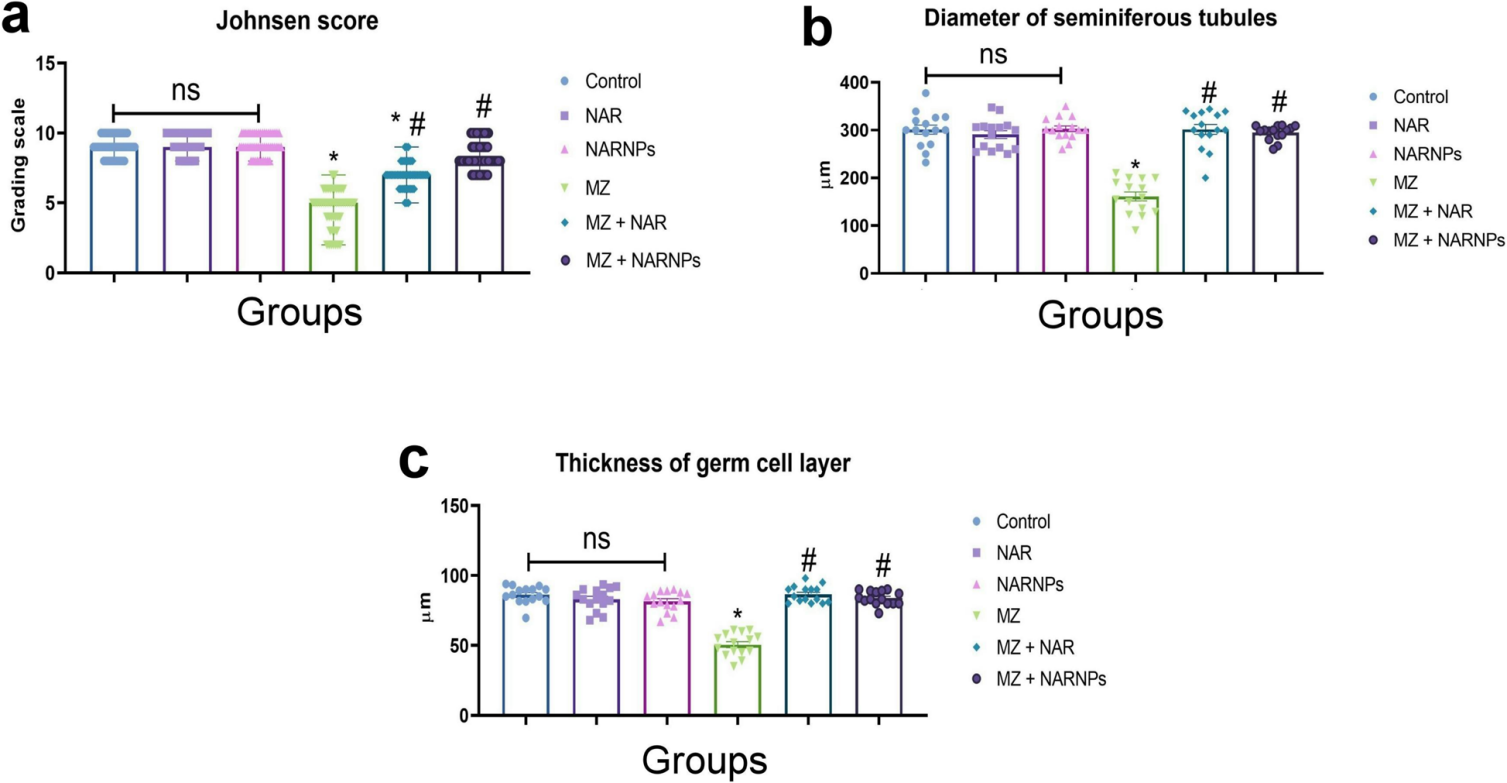
Scatter plot bar represents the mean testicular Johnsen score (**a**), seminiferous tubular diameter (**b**), and germ cell thickness (**c**) in various experimental groups. Values presented as median with range for Johnsen score (n = 50 ST/group) and mean ± SEM for other measurements (n = 15 ST/group). ⁎ means a significant difference compared to the corresponding control group, while # means a significant difference compared to MZ group at *P* ≤ *0.05*

Johnsen score recorded the lowest score in the MZ group, while the highest score was noticed in the NARNPs group which resembled the control group. The average score of the NAR group was significantly higher than that of the MZ group, but still low if compared with the corresponding control group. Moreover, the MZ group showed a significant reduction in the seminiferous tubule diameter and germ cell layer thickness if compared with the control group. Otherwise, the co-treatment of NAR, whether in free form or nano-formulation, with MZ significantly improved all the histomorphometric parameters compared to the MZ group (Fig. 4).

### Immunohistochemical localization of some proliferating markers within testes

**Fig. 5:**
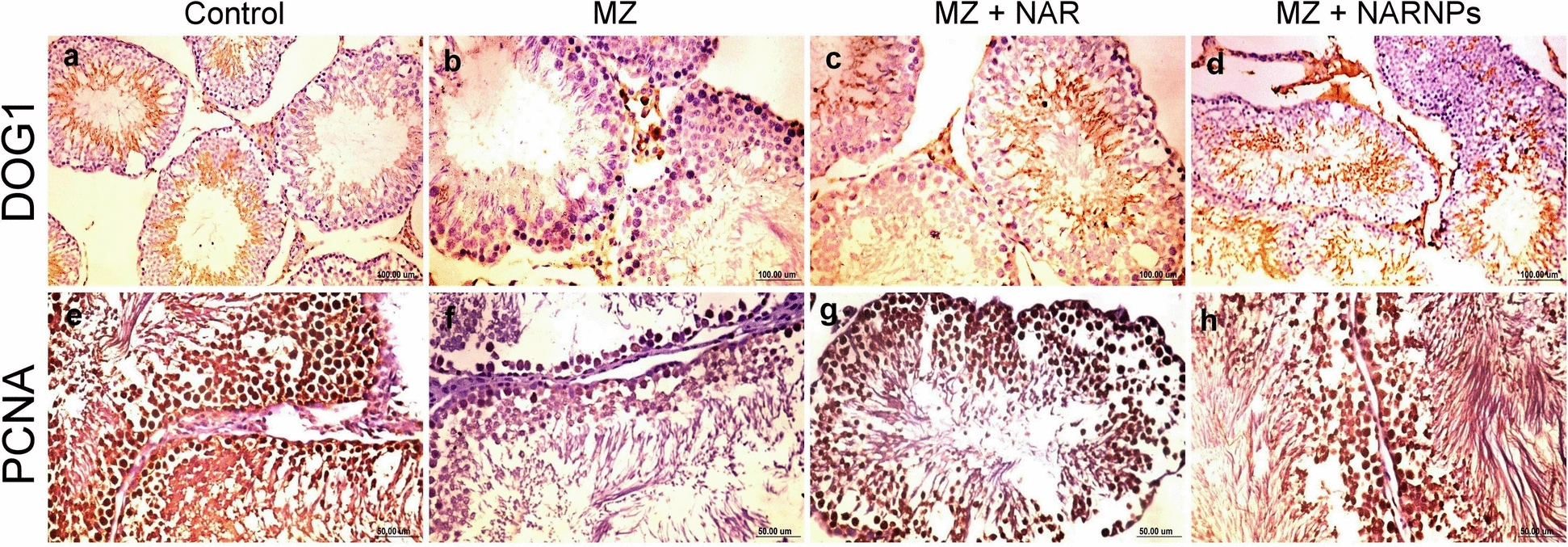
Photograph represents DOG1 and PCNA localization within testicular sections of various experimental groups. **a**, **e** The control group showed strong DOG1 and PCNA immunoexpression. **b**, **f** MZ group showed weak DOG1 and PCNA immunoexpression. c, g MZ + NAR group and d, h MZ + NAR NPs group both showed strong DOG1 and PCNA immunopositivity. Scale bar 100 µm corresponding to 10 × magnification while scale bar 50 µm corresponding to 20 × magnification

**Fig. 6:**
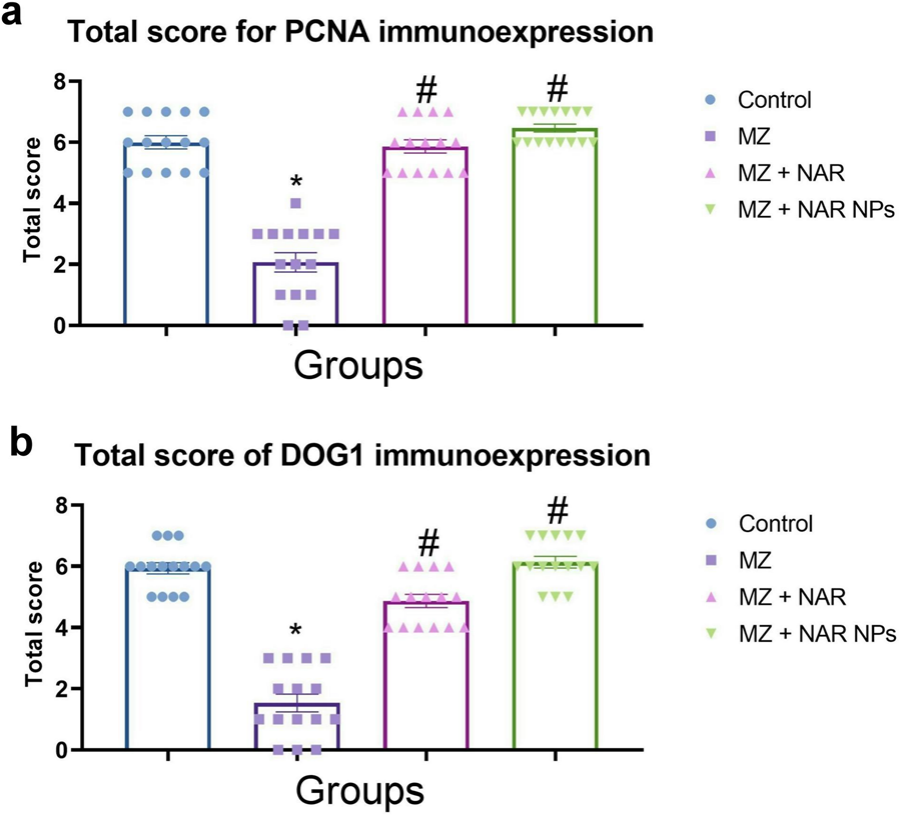
Scatter plot bar represents the total score of both PCNA (**a**) and DOG1 (**b**) immunoexpression in testes of various experimental groups. Values presented as median with range (n = 15 ST/group). ⁎ means a significant difference compared to the corresponding control group, while # means a significant difference compared to MZ group at *P* ≤ *0.05*

The proliferating markers (PCNA, DOG1) were expressed slightly in the MZ group but strongly expressed in both NAR-treated groups. Whereas the control group showed strong positive expression of both proliferation markers. The highest total score was recorded in the NARNPs group, followed by the NAR groups, which resembled the control group. The lowest total score of both proliferating markers was detected in the MZ group (Figs. 5 and 6).

## Discussion

**Fig. 7:**
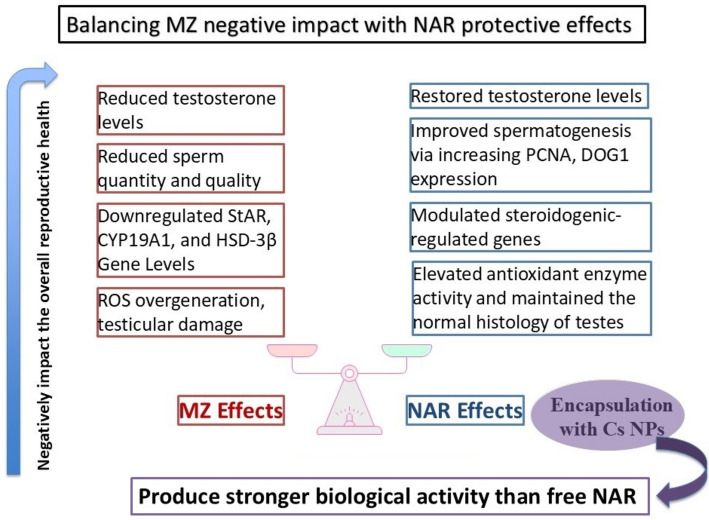
Associated evidence underlying the protective role of NAR and Cs-coated NARNPs against MZ-induced reproductive toxicity. The daily oral administration of MZ increased ROS overgeneration and decreased antioxidant enzyme activities in testes, inducing testicular damage manifested by reducing germ cell thickness, damaging Sertoli cells and Leydig cells, and reducing testosterone levels. MZ downregulated the transcriptase levels of *the CYP19A1* gene that negatively impacted sperm production and maturation, interfering with the process of spermatogenesis. It also downregulated *StAR* and *HSD-3β* gene levels, causing decreased testosterone levels, impacting both spermatogenesis and overall male reproductive health. The coadministration of NAR with MZ moderately improved the sperm quality parameters, partially restored the testosterone levels, and maintained the histology of testicular tissue via reducing ROS production, elevating antioxidant activity, modulating steroidogenic regulated genes, and increasing the expression of proliferating markers PCNA and DOG1 in germ cells. Otherwise, the NARNPs produced a stronger protective effect than free NAR but did not fully normalize all measured endpoints

Pesticides are widely employed in agriculture to manage dangerous pests and avoid crop output losses or product damage. Human exposure to pesticides is increased by their prolonged environmental persistence, particularly for farmers who use them often [1]. It is important to find novel strategies to avoid pesticide toxicity in humans and livestock. Therefore, the present study aimed to explore the possible protective effect of naringin, either free or nano-formulation, against reproductive toxicity of mancozeb in adult male rats, as illustrated in Fig. 7. Our findings revealed that the daily exposure to MZ significantly decreased testosterone levels, altered both sperm quality and quantity, and induced testicular damage. A significant decline in testosterone levels is attributed to the toxic impact of MZ on Leydig cells, which are responsible for testosterone production. This observation has been proved by our histopathological examination, which demonstrated degeneration and necrosis of interstitial Leydig cells. Additionally, there were extensive desquamation and loss of germinal epithelium, irregularity, and atrophy of seminiferous tubules. In agreement with our findings, Moreira et al. [59], revealed a degeneration of Leydig cells and a disruption of the germinal epithelium in the testes of MZ-exposed rats, which is in accordance with a significant decrease in serum testosterone levels. It was reported that chronic exposure to MZ resulted in decreased testosterone concentrations and oxidative stress-mediated histopathological alterations in testicular tissue [50]. Moreover, our results were accompanied by substantial impairments in the sperm quality parameters, including reduced motility, viability, and sperm concentration, as well as increased abnormalities, which were consistent with Kumawat et al., [49], who reported these sperm alterations in rats following oral administration of MZ at 500 mg/kg for 28 days.

The outcomes of the current study revealed that oxidative stress plays a vital role in MZ-induced reproductive toxicity manifested by elevated MDA levels and reduced GR and catalase activity in the MZ group. MDA is considered a strong biomarker of lipid peroxidation, reflecting enhanced reactive oxygen species (ROS)-mediated damage to cellular membranes [37]. Moreover, the diminished activities of GR and catalase suggest a compromised antioxidant defense system. GR is essential for keeping intracellular levels of reduced glutathione (GSH), a major non-enzymatic antioxidant, while catalase plays a critical role in decomposing hydrogen peroxide into water and molecular oxygen [2]. The impairment of these enzymatic defenses, reflecting redox imbalance, contributes to testicular cellular damage, impaired spermatogenesis, and endocrine disruption following MZ exposure [12, 19].

From the molecular insight, the MZ group showed downregulation of major steroidogenic regulating genes, including *Cyp19a1, Hsd-3β,* and *StAR.* StAR gene is a key regulator of cholesterol transport into mitochondria, which is needed for testosterone biosynthesis [33]. This result supports an explanation for the MZ-induced lowering in testosterone levels in the present study. Additionally, the downregulation of both CYP19A1 and HSD-3β genes could disturb both estrogen production and local paracrine signaling, which are critical for both germ cell survival and testosterone biosynthesis [24]. Several published studies agreed with our findings about the suppressive effect of MZ on the testicular endocrine axis [50, 75]. Additionally, the immunohistochemical staining showed weak PCNA and DOG1 immunostaining in the testes obtained from the MZ group in response to testicular damage and disrupted steroidogenesis [13]. It has been reported that PCNA and DOG1 are considered as proliferating markers of spermatogenesis, indicating germ cell maturation. PCNA is expressed in the nucleus of the proliferating spermatogonia and primary spermatocytes [73]. While DOG1, a calcium-activated chloride channel, is expressed in the cytoplasmic membrane of secondary spermatocytes and spermatids and is absent from spermatogonia [69].

The current data proved that the co-treatment with NAR, particularly nanoformulation, significantly elevated the testosterone levels and improved sperm quality parameters compared with the MZ group. Moreover, the MDA level was significantly reduced along with increasing of both antioxidant enzyme activities, suggesting the antioxidant potential of NAR. Histological analysis showed significant recovery of testicular structure alongside improvement in Johnsen score; however, it did not fully return to the control level in the NAR treatment group. Additionally, NARNPs produced a stronger protective effect than free NAR, but did not fully normalize all measured endpoints. The encapsulation of NAR with Cs NPs significantly improved its biological activity and showed superior improvement. The anti-toxic effect of NAR was recently investigated in many studies, which may be matched with our findings [7, 51]. A recent study investigated the cytoprotective effect of orally administered NAR against Di-n-butylphthalate-induced testicular damage, which significantly increased serum testosterone levels and improved both sperm quantity and quality [14]. Furthermore, oral administration of NAR significantly restored testosterone levels and the antioxidant status, suggesting its protective role against Bisphenol A-induced male reproductive toxicity [10]. Recent studies linked the protective effects of NAR against testicular damage with the potent antioxidant effect of NAR [8, 63]. The antioxidant properties of NAR could clearly contribute to reducing the extent of testicular damage induced by MZ and preserving the testicular cell integrity.

NAR seems to be protected against MZ-induced testicular damage, apparently through several coordinated mechanisms, including promotion of cell proliferation in the damaged tissue [14]. Moreover, NAR treatment in many rodent models of reproductive toxicity could upregulate PCNA and DOG1, suggesting the reactivation of proliferating pathways [10, 69]. Mechanistically, the antioxidant effect of NAR is achieved by scavenging free radicals and restoring redox enzyme activity, which reduces oxidative stress and makes the environment safe and stable for spermatogenic cells to repair and multiply [77]. Higher sperm counts, better hormonal balance, and improved testicular histology are all attributed to this proliferative boost. Moreover, *StAR, CYP19A1* and *HSD-3β* gene expressions showed a significant upregulation in both NAR + MZ and NARNPs + MZ groups, suggesting the role of NAR in improving spermatogenesis and steroidogenesis.

The improved outcomes in the NARNPs group may be attributed to the combined effect of NAR and Cs NPs. NARNPs were successfully prepared and fully characterized by several methods, including XRD, Zeta sizer nano, and TEM. XRD analysis was performed to investigate the crystalline structure and phase changes of Cs NPs and NAR NPs. The characteristic broad peak is indicative of semi-crystalline nature of Cs NPs, arising from its polymeric structure and intermolecular hydrogen bonding [30], while the sharper peaks are characteristic of the crystalline nature of NAR [54]. The appearance of these peaks in the NAR NPs suggests that NAR keeps its crystalline form and is partially encapsulated within the Cs matrix without undergoing complete amorphization. The reduction in the intensity and broadening of the chitosan peak, coupled with the appearance of NAR’s crystalline peaks, indicates a successful interaction and integration of NAR within the Cs NPs structure, potentially leading to a slight disruption of the chitosan’s inherent semi-crystalline order [65]. This observation is crucial as it confirms the presence of NAR in its crystalline state within the nanocomposite, which can influence its stability and release characteristics.

The hydrodynamic diameter and polydispersity index (PDI) are critical parameters for characterizing nanoparticles, providing insights into their size distribution and homogeneity [74]. Our results indicated an increase in the size of NAR NPs compared with Cs NPs, suggesting the successful encapsulation of NAR with the Cs NPs. The PDI value still falls within the acceptable range (typically < 0.3–0.5) for pharmaceutical applications, indicating a relatively homogeneous population of the nanocomposite particles [66]. The increase in particle size and PDI after loading is a common phenomenon observed in drug delivery systems, attributed to the incorporation of the active compound and potential changes in the particle’s hydration layer [61]. Zeta potential is a crucial indicator of the surface charge and colloidal stability of nanoparticles. Our results proved the characteristic positive charge of Cs NPs, owing to the protonation of its amine groups in acidic or neutral environments, which contributes to electrostatic repulsion among particles, thereby enhancing their colloidal stability [22]. Moreover, this further positive shift in zeta potential after NAR incorporation suggests that the surface charge characteristics of the Cs NPs are largely maintained, possibly due to the surface exposure of positively charged groups from NAR or its interaction with Cs. A high positive zeta potential, generally above + 30 mV or below -30 mV, is indicative of good colloidal stability, preventing aggregation of the nanoparticles in suspension [52]. The sustained positive charge of the nanocomposite is beneficial for interactions with negatively charged biological membranes, which are often desired for drug delivery applications [62].

The TEM analysis offers crucial insights into the physical characteristics of NAR NPs. The observed spherical morphology is consistent with many reports on chitosan-based nanoparticles prepared by ionic gelation, a common method for encapsulating bioactive compounds [9]. This method typically involves the electrostatic interaction between the positively charged amino groups of chitosan and negatively charged crosslinking agents, TPP, leading to the formation of stable nanoparticles [36]. The spherical shape is generally desirable for drug delivery systems as it can help with cellular uptake and improve biodistribution [81, 82]. Furthermore, the estimated particle size range of 70–150 nm aligns well with the best size range for various biomedical applications, particularly for drug delivery and targeting. Nanoparticles within this size range are known to effectively bypass biological barriers, accumulate in target tissues through the enhanced permeability and retention (EPR) effect [21]. The relatively uniform size distribution seen in the TEM image suggests a controlled synthesis process, which is critical for reproducible therapeutic outcomes and minimizing batch-to-batch variability. The absence of significant aggregation in the TEM image indicates good colloidal stability of the NAR NPs, which contributed to increased circulation time, biodistribution, and cellular uptake. The positive surface charge of chitosan nanoparticles, often reflected in their zeta potential, contributes to their stability by providing electrostatic repulsion among particles, thereby preventing agglomeration [20].

Our results also showed high EE and LC for the prepared NAR NPs, indicating a successful encapsulation. This may be attributed to the strong electrostatic interactions between the cationic amino groups of the Cs backbone and the anionic or polar functional groups of the NAR molecule, supplemented by hydrogen bonding and hydrophobic interactions within the polymeric matrix [43]. Furthermore, the LC of 11.58% proves that a large amount of NAR was successfully incorporated into the nanoparticles. This level of drug loading is therapeutically promising, as it suggests that a sufficient quantity of the active compound can be delivered to the target site, potentially reducing the overall dosage required and minimizing off-target effects. The balance between a high EE and a significant LC is often challenging to achieve; a high EE does not always guarantee a high LC, as the latter is also dependent on the first drug-to-polymer ratio and the intrinsic properties of the drug and carrier. Therefore, the values obtained in this study represent successful optimization and the creation of a potent drug delivery vehicle.

These findings suggested that the superior biological effects of NARNPs may be related to enhanced bioavailability, improved cellular uptake, controlled release, and delivery to the target site. Many recent studies agreed with this theory, while they found that nanoformulated medicinal plant extracts can effectively reduce toxicity and promote recovery in animal models [64, 71]. One study investigated that Cs NPs encapsulation could increase the antioxidant and anti-apoptotic effects of curcumin against nickel-induced hepatorenal damage in rats by increasing its oral absorption and bioavailability [37]. Another study revealed that the encapsulation of propolis extract by Cs NPs could improve steroidogenesis and spermatogenesis, enhance testicular antioxidant protection, and potentially reduce the risk of diabetes better than propolis [31]. Meanwhile, it was proven that the encapsulation of quercetin with Cs NPs significantly enhances its antioxidant, anti-inflammatory, and anti-apoptotic activities [3]. Additionally, many recent studies confirmed the protective role of several nanoformulations such as selenium nanoparticles, quercetin nanoparticles and thymoquinone-loaded chitosan nanoparticles against testicular toxicity, confirmed by hormonal gene expression and other parameters (Suhas et al*.,* 2024; [47]).

Although the current study provides insightful information on the protective role of NAR and Cs-coated NARNPs against MZ-induced reproductive toxicity, some limitations should be acknowledged. First, our findings mainly demonstrate association, rather than direct mechanistic confirmation, based on biochemical, hormonal, histological, and immunohistochemical assessments. Further targeted experiments are required to validate these mechanisms. Second, the enhanced efficacy of the nano-formulation is suggested by the biological outcomes, but a lack of pharmacokinetic and tissue distribution data prevents definitive conclusions about its superiority. Third, the model reflects sub-chronic exposure to MZ, unlike many chronic and mixed-toxicant human exposures which require further investigations. Finally, the study focused on male reproductive toxicity, as it does not address female reproductive toxicity or cross-species variability. Further research should include female models to investigate sex-specific variations in susceptibility and outcomes.

## Conclusion

The current study concluded that the MZ exposure leads to a significant reproductive toxicity in male rats, evidenced by reduced sperm quality and quantity, lowered testosterone levels, elevated oxidative stress markers, and downregulation of the key steroidogenic genes. The co-administration of NAR with MZ significantly alleviated such toxicity, but the nano-formulations (NARNPs) offered more pronounced improvements. The administration of NARNPs with MZ partially restored male sex hormonal levels, enhanced antioxidant activity, improved sperm parameters, and upregulated steroidogenic gene levels. Histological and immunohistochemical findings further confirmed better testicular structure and increased cellular proliferation in the NARNPs group. These results revealed that chitosan-coated NARNPs are more effective than free NAR in mitigating testicular toxicity and promoting reproductive recovery.

## Data Availability

All data will be made available on request.
